# Urban soil pollution in Türkiye: a review of potentially toxic elements, polycyclic aromatic hydrocarbons, and microplastics in major cities

**DOI:** 10.1007/s10661-025-14834-5

**Published:** 2025-11-24

**Authors:** Gülfem Çayır, Abdulmannan Rouhani, Karim Suhail Al Souki, Lidia Błażałek, Robert Ato Newton, Fatma Zehra Şükür, Valentina Pidlisnyuk

**Affiliations:** 1 Department of Environmental Engineering, Civil Engineering Faculty, Davutpasa Mah. YTU Davutpasa Campus, Yildiz Technical University, Istanbul, 34220 Türkiye; 2 Department of Environment, Faculty of Environment, Jan Evangelista Purkyně University in Ústí nad Labem, Pasteurova 15, 400 96 Czech Republic; 3 Department of Environmental Chemistry and Technology, Faculty of Environment, Jan Evangelista Purkyně University in Ústí nad Labem, Pasteurova 15, 400 96 Czech Republic; 4 Department of Plant Physiology and Biochemistry, Faculty of Biology and Environmental Protection, University of Lodz, Banacha Str. 12/16, 90-237 Lodz, Poland

**Keywords:** Urban soil contamination, Anthropogenic activities, Pollution monitoring, Urbanization, Türkiye

## Abstract

Rapid urban development and industrialization have led to severe soil pollution, which has become a major global environmental problem, further intensified by population growth and land-use changes. In urban soils, pollution from past anthropogenic activities can still be detected through its harmful effects on soil quality, ecosystems and human health. This review summarizes the status of potentially toxic elements (PTEs), polycyclic aromatic hydrocarbons (PAHs), and microplastics (MPs) in Türkiye’s urban soils. Across cities, Cd and Pb frequently exceeded national soil-quality thresholds, with industrial hubs and traffic corridors emerging as hotspots. PAH burdens often reached the 'heavily contaminated' category (> 1000 µg kg⁻^1^), with winter maxima linked to heating activities and a dominance of pyrogenic four-ring compounds. Urban MPs were abundant in parks and along roadsides, dominated by fibers and polyethylene, and showed strong variation with land use (recreational > industrial > residential in İstanbul). This evidence illustrates a lack of in-depth understanding of the full extent of soil pollution in targeted areas. The results of this review will help develop practical strategies for soil management, pollution monitoring, and remediation, which will ultimately improve public health and support sustainable development in Türkiye.

## Introduction

Urban soil pollution has increased in recent years due to population growth and urban economic development, posing a substantial risk to human health and well-being (Tang et al., 2024). Currently, Türkiye has been recognized as a thriving commercial and industrial center with growing economic activities. This refers primarily to large metropolises such as Bursa, İzmir, and Istanbul, which over the last decades have undergone rapid urban expansion. Consequently, these advances led to rapid and one-way migration from rural to urban regions (Sağlam, 2016). Thus, in the early 1950 s, only 15% of the Turkish population settled in urban area. However, this ratio rose to 31.5% in 1960, 38.2% in 1970, 43.8% in 1980, and eventually reached 64.7% by 2000 (Khorrami et al., 2021). Türkiye has a population of 85,372,377, ranking 18th among 194 countries in population value, which constitutes 1.1% of the global total population. Despite its increasing economic prosperity, the country is currently experiencing environmental degradation due to limited attention to environmental conditions, low public environmental awareness, and inadequate or weakly implemented government regulations. All of these factors has resulted in an unplanned urbanization process (Udemba & Keleş, 2022).

Urban soils have a limited capacity for pollutant absorption and natural self-purification. Combined with urban sprawl and industrial expansion, this limitation has intensified the release of potentially toxic elements (PTEs) into the environment (Swain, 2024; Tang et al., 2024). The entry of PTEs into urban soils occurs mainly through two pathways: natural processes and human-driven activities (Shi et al., 2024). Natural inputs arise largely from the characteristics of parent material and soil-forming processes, whereas anthropogenic inputs originate from fossil fuel combustion, waste burning, traffic emissions, metal processing, and intensive agricultural practices (Adnan et al., 2024; Rouhani et al., 2023a; Simatupang et al., 2024). The accumulation of PTEs in soils disrupts ecological functions and biogeochemical cycles, causing issues such as soil degradation and alterations in its structure and properties (Dondini et al., 2024; Gopal et al., 2024). Furthermore, these elements can accumulate in the human body in metallic or compound forms, potentially leading to serious physiological and biological disorders (Moukadiri et al., 2024; Rouhani et al., 2022).

Polycyclic aromatic hydrocarbons (PAHs) are hazardous substances that can cause cancer and are recognized for their significant hydrophobic properties and resilience against biological degradation. They are substances consisting of two or more linked benzene rings, with a reduction that is approximately linear as the molecular weight increases (Fu et al., 2024; Kuppusamy et al., 2017). The main source of PAHs is incomplete combustion processes such as domestic cooking, gasoline combustion in vehicles, and the use of coal in residential and industrial sectors (Hümmler et al., 2024; Peng et al., 2011). In particular, one of the main sources of PAHs in urban soils is the exhaust from motor vehicles (Akinsete & Olatimehin, 2024; Morillo et al., 2007). Atmospheric deposition and street dust are also important sources of PAHs in surface soil (Rouhani et al., 2024). Accumulated PAHs in soil can contaminate plants and food chains, which in turn can pose direct and indirect risks to human health (Moon et al., 2024; Zohair et al., 2006). PAHs can indue carcinogenic, mutagenic, and teratogenic effects in humans and other species (Venkatraman et al., 2024).

Plastic is commonly used in various industrial and consumer sectors due to its cost-effectiveness, light weight, and durable and resilient characteristics (Bian et al., 2022). Global plastic production has reached about 400 million tonnes worldwide, showing a slight yearly increase, with China accounting for 32%, North America 17%, and Europe 14% of the total (Plastics Europe, 2023). In developing countries, only a small fraction of plastic waste is recycled, while most is disposed of in landfills. Once in landfills, plastic waste is exposed to sunlight, microbial activity, and varying atmospheric conditions (Dehghani et al., 2017; Rouhani & Hejcman, 2024). Moreover, it undergoes a variety of processes in landfills, including mechanical abrasion, thermo-oxidative and thermal deterioration, photo-degradation, and biodegradation. As a result, these processes generate and release microplastics (MPs) into urban soil and road dust. Moreover, MPs can be easily dispersed by wind and vehicular movement, resulting in their re-suspension from landfills and urban surfaces (Jaafarzadeh & Talepour, 2024; Rouhani et al., 2024). MPs are tiny plastic fragments that have the potential to accumulate, migrate, and disperse within the environment. This results from their stable chemical properties, large surface area, small size, and hydrophobic nature (Chukwuemeka et al., 2024). MPs can significantly impact soil by changing its pH, bulk density, and reducing water-holding capacity. These changes in soil can subsequently have negative effects on animals, microbes, plant growth and physiology, and human health (Irshad et al., 2024; Liu et al., 2024). MP exposure is suspected to negatively affect human reproductive, digestive, and respiratory health. Recent findings indicated connections to sperm quality reduction, gut immunosuppression, lung injury, and potential associations with colon and lung cancers (Chartres et al., 2024). In addition to their inherent toxicity, MPs can serve as carriers for other contaminants, including PTEs and PAHs found within soils (Hildebrandt et al., 2021; Hu et al., 2022).

Although global attention to urban soil contamination has increased, research on this issue in Türkiye remains limited, especially with respect to MPs and PAHs. While several reviews have examined urban soil pollution at global and national levels, to our knowledge no comprehensive review has specifically focused on Türkiye. In addition, most existing reviews emphasize individual pollutants rather than addressing the coexistence of multiple contaminants. This review attempts to address these gaps by providing an integrated assessment of urban soil pollution in Türkiye, with particular attention to the occurrence, sources, and impacts of MPs, PAHs, and PTEs. By analyzing these pollutants collectively, the present study enhances understanding of urban soil contamination patterns and sources in Turkish cities. Its novelty lies in the holistic evaluation of diverse contaminants and their interlinked sources, advancing knowledge essential for managing environmental and public health risks associated with polluted urban soils in Türkiye. Utilizing this multi-contaminant perspective contributes to the field and supports targeted efforts to mitigate urban soil pollution through improved management and remediation strategies.

## Methodology

This review was conducted to identify and emphasize the presence of PTEs, PAHs, and MPs as pollutants in urban soils of Türkiye. The review method involved collecting articles from the literature databases, screening them, and discussing the selected relevant studies. The sources of literature were obtained from two databases using the following keywords: soil, contamination, urban, and Türkiye. Consequently, the search was conducted on the Web of Science (WOS) and Google Scholar (GS) using Soil* AND (Contamination OR Pollution) AND Urban* AND (Turkey OR Türkiye). A total of 167 results were obtained from the WOS.

### Screening of literature

For this review, a thorough screening process was applied to the initially collected articles. Selection followed several key criteria: language (English only); document type (peer-reviewed research articles, excluding reviews and books concerning soil pollution in Türkiye); and topical relevance (studies addressing target soil pollutants, PTEs, PAHs, and MPs, in urban areas of Türkiye). In total, 267 publications were retrieved from the WoS and GS databases and subsequently screened through multiple stages:

- Language exclusion: all retrieved papers were in English, so no exclusions were made based on this criterion.
- Document type exclusion: review articles, books on soil pollution in Türkiye lacking case studies, and non-peer-reviewed conference papers were excluded, resulting in the removal of 12 publications.
- Title filtering: titles of peer-reviewed articles were screened to exclude those clearly outside the review's scope. Articles discussing air or water pollution, mining or non-urban areas, soil remediation, or other countries besides Türkiye were excluded, eliminating 144 articles.
- Abstract selection: abstracts of the remaining articles were reviewed, and those addressing non-urban areas in Türkiye or pollutants other than PTEs, PAHs, and MPs were excluded. This step removed 39 articles.
- Full-article screening: for articles with unclear abstracts regarding the location of soil pollution or the type of pollutants, the entire paper was read. Articles that did not meet the review’s criteria were excluded, resulting in the removal of 32 articles.
- Duplicate exclusion: a total of 14 duplicate articles found in both the WOS and GS databases were removed.

**Fig. 1:**
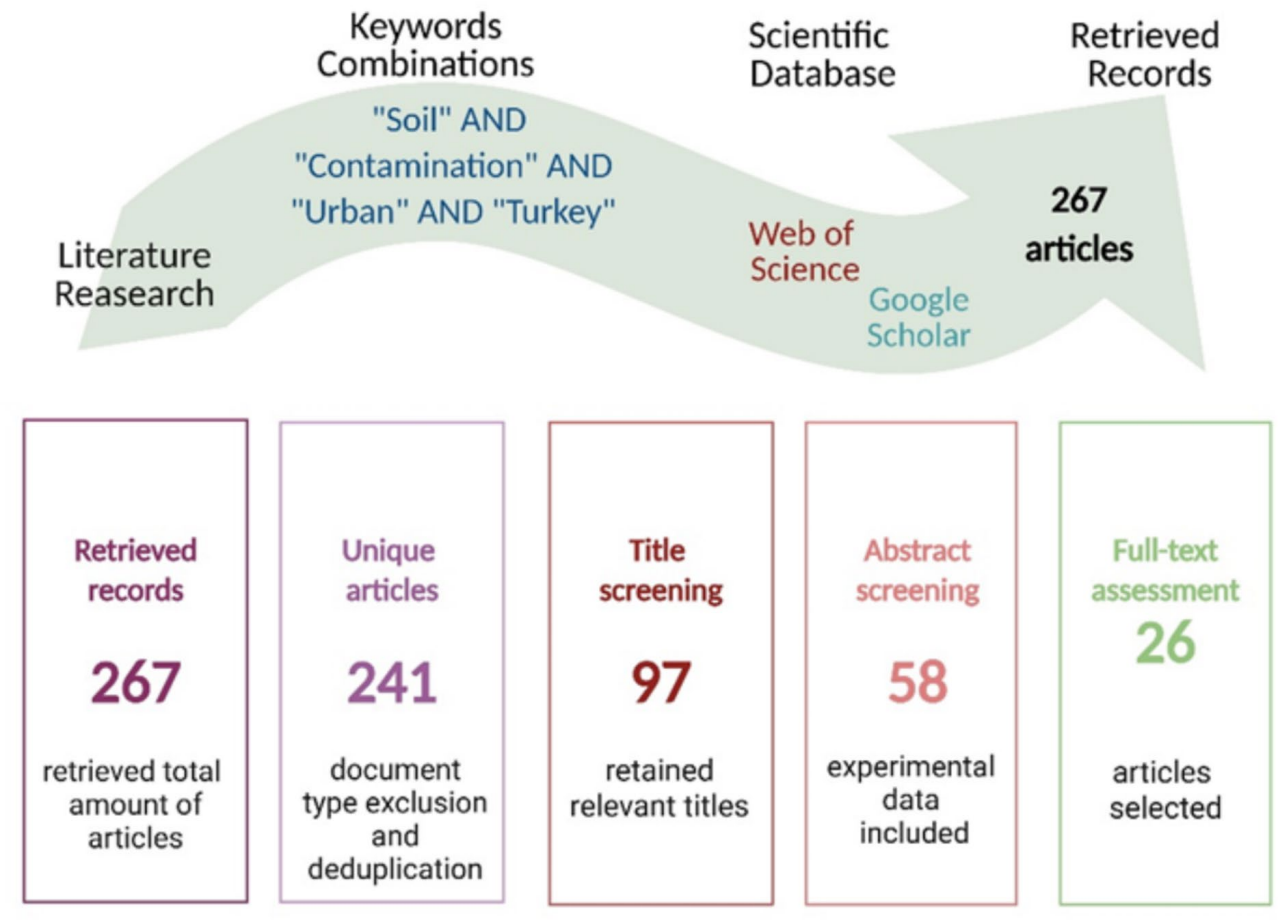
Flowchart showing the literature search and screening process

Following this comprehensive screening process, 26 articles were selected for incorporation in the review. Among these, 16 articles covered PTEs pollution, 7 articles addressed PAHs pollution, and 3 articles discussed MPs contamination in urban soils in Türkiye. Figure 1 presents the methodological procedure for literature search and screening.

## Urban soil

Urban soils, as integral components of urban ecosystems, influence environmental quality and human health. Although they maintain some properties of their natural parent materials, urban soils are substantially modified by high population density and continuous anthropogenic activities (Tang et al., 2024). In urban areas, soil horizons are often irregular, characterized by anthropogenic deposits and significant heterogeneity caused by human disturbances and infilling. Many factors, including human trampling, mechanical compaction, artifacts, and technogenic substrata, contribute to the deterioration of soil structure (Greinert, 2015; Yang & Zhang, 2015). These soils are characterized by high contamination level, low water content, poor permeability, and unpredictable stratification (Napoletano et al., 2021).

Rapid urbanization, along with industrialization, leads to increased pollutant inputs in urban areas, owing to the limited environmental carrying capacity and restricted self-purification potential of urban systems. Such inputs place substantial pressure on biogeochemical cycles and urban ecosystems. This pressure contributes to environmental challenges such as the degradation of soil functions and modifications in soil structure and properties (Yang et al., 2024; Zhu et al., 2024). Consequently, the capacity of urban soils to deliver ecosystem services is significantly threatened by excessive pollutant loading that diminishes ecological resilience. Once the environmental threshold is exceeded, urban soils may shift from functioning as pollutant sinks to becoming secondary sources of contamination, creating long-lasting environmental risks (Li et al., 2018; Yang & Zhang, 2015).

Despite substantial disturbances, urban soils can still support plants, animals, and microorganisms, while also regulating biogeochemical and hydrological cycles in ways comparable to rural soils. The soil biota in urban environments thus represents a distinct assemblage, consisting of native species that persist or adapt under urban conditions together with non-native species introduced from other regions or continents (Pouyat et al., 2020).

## Urban soil pollution in Türkiye

### Potentially toxic elements

Pollution by PTEs is a major environmental concern that poses serious risks to human health and has been attracting increased global attention, particularly in urban areas (Akinsete & Olatimehin, 2024; Bartkowiak et al., 2024; Mahvi et al., 2022; Răcușan Ghircoiaș et al., 2023). PTEs are recognized as key contributors to urban pollution, with urban soils and dust functioning as their primary reservoirs and secondary sources through processes such as resuspension and leaching. These contaminants mainly originate from anthropogenic emissions such as traffic, fossil fuel combustion, and industrial activities. Continuous inputs from such sources have led to the accumulation and long-term persistence of PTEs in urban soils, even after direct emission points are removed. Consequently, PTE-enriched urban soils represent sensitive indicators of overall urban environmental quality (Li et al., 2020; Pavlović et al., 2021; Rouhani et al., 2023b).

**Fig. 2:**
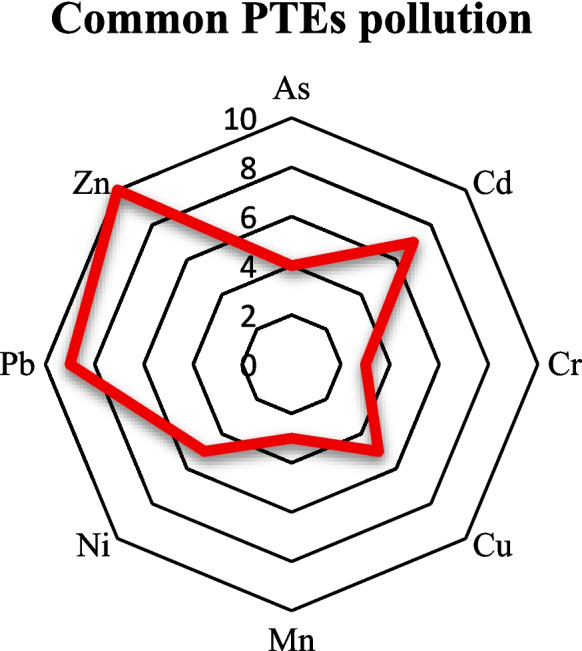
Frequency of occurrence of PTEs with high pollution levels in urban areas across Türkiye

Table 1 and Fig. 2 show that in various Turkish urban areas, Zn is the most prevalent PTEs pollutant, followed by Pb and Cd, respectively, of other investigated PTEs. PTEs with high pollution levels were identified based on the classifications reported in the original studies, typically referring to elements exceeding national or local soil-quality standards or showing the highest concentrations within the dataset. Other notable PTEs identified in the soil of urban areas in Türkiye are As, Cr, Cu, Mn, and Ni.

**Table 1:** Soil pollution by PTEs in Turkish urban areas

City	Analyzed PTEs	PTEs with high pollution level	References
Ömerli,İstanbul	Br, Cr, Mn, Ni, Pb, Ca, P, Zn	Pb, Ni	Esen et al. (2024)
Uşak	As, Cr, Cu, Hg, Ni, Pb, Cd, Zn	As, Cr, Ni	Yildiz and Ozkul, (2022)
Tuzla, İstanbul	Cu, Pb, Zn, Hg, Cd	Zn, Cu, Pb	Sezgin et al. (2019)
Batman	Co, Ni, Cu, Zn, Ga, As, Mo, Sn, Sb, Ce, Pb, U	As, Mo, Sb	Baran and Gumus Kiral, (2021)
Bingöl	Fe, Cd, Pb, Cr, Mn, Co, Ni, Cu, Zn	Zn, Pb, Cd, Ni	Vural et al. (2021)
Muğla	Pb, Cd	Cd	Demirak et al. (2022)
Kütahya	As, Hg, Sb	As, Sb	Guney et al. (2020)
Denizli	Fe, Cr, Ni, Zn, Cu, Pb	Cu, Pb, Zn	Oudeika et al. (2020)
Gebze,İstanbul	Cd, As, Pb, Zn, Mn, Cu, Cr, Hg	Cd, As, Pb, Zn, Mn, Cu, Cr	Yaylalı-Abanuz (2011)
Ordu	Cu, Ni, Pb, Zn, Cr, Cd	Zn, Cd, Pb	Yesil and Yesil, (2019)
Kahramanmaraş	Ni, Pb, Zn, Cu, Fe, As, Cd	Pb, Zn, Cd	Ezer (2009)
Mersin	Pb, Ni, Cd, Cu, Zn	Pb, Zn, Cd	Arslan and Gizir, (2006)
İzmit	Co, Cu, Mn, Ni, Pb, Zn, Cr, Fe	Co, Cu, Mn, Ni, Pb, Zn	Yilmaz et al. (2003)
İstanbul	Ba, Rb, Ce, La, Th, Sc	Ba, Rb	Haciyakupoglu et al. (2014)
Eskişehir	As, Cr, Cd, Co, Cu, Ni, Pb, Zn, Fe, Mg	Cr, Cu, Ni, Zn, Cd	Malkoc and Yazici, (2017)
Hatay-Iskenderun	As, Cd, Cr, Cu, Ni, Pb, Sn, Zn	Zn, Pb	Odabasi et al. (2010)

Esen et al. (2024) reported extreme Pb and Ni contamination in Ömerli, Istanbul, where prolonged exposure posed a non-carcinogenic threat to children. Sezgin et al. (2019) examined soils in the Tuzla district (located adjacent to an airport, industrial site, and racetrack) and found PTE concentrations in the order of Zn > Cu > Pb > Cd. Except for Hg, the mean concentrations exceeded the average crustal values. However, the ecological risk assessment suggested a moderate to unpolluted state, indicating limited influence from the nearby airport and racetrack. Mahvi et al. (2022) similarly reported low levels of PTEs around an airport in Kerman, Iran, suggesting that aviation-related soils are not uniformly contaminated across sites. Yesil and Yesil (2019) observed high Cd, Cr, Ni, Pb, and Zn concentrations above threshold values in playground soils of Ordu, where traffic and industrial emissions were the dominant sources. Similar contamination has been reported in playgrounds worldwide, Pb in Accra, Ghana (Kyene et al., 2023), Cd in Bandar Abbas, Iran (Ghaffari et al., 2023), and Zn and Pb in Cluj-Napoca, Romania (Răcușan Ghircoiaș et al., 2023).

Vural et al. (2021) found that soils in Bingöl's residential and industrial zones were most contaminated, with Cu, Co, Cr, Fe, Mn, and Pb elevated in residential areas, while Zn was highest in commercial and industrial districts. Traffic near highways was the dominant source, while Cd was associated with improperly disposed wastes (batteries, plastics, cans). Demirak et al. (2022) analyzed Pb and Cd fractions in Muğla city soils and identified Cd as the primary risk factor, with concentrations exceeding permissible limits in multiple locations. Main sources were traffic emissions, construction excavation soils, and irrigation with wastewater from municipal treatment plants, all of which contributed to Cd enrichment. Yaylalı-Abanuz (2011) reported extremely high Cd, Pb, Zn, Mn, Cu, As, and Cr in Gebze's industrial soils, reflecting decades of uncontrolled hazardous waste disposal and heavy traffic. Comparable levels of As, Cu, Pb, and Zn were recorded in Osogbo, Nigeria (Kolawole et al., 2023). Haciyakupoglu et al. (2014) found elevated Ba and Rb in industrialized soils near Istanbul, attributable to rapid urbanization and industrialization. Malkoc and Yazici, (2017) documented Cd, Cu, and Zn contamination in surface soils at Eskişehir Organized Industrial Site. Oudeika et al. (2020) reported high Pb levels in Denizli city soils, primarily linked to industrial activities and transportation. The large industrial zone and intensive cargo traffic via road and rail contributed substantially to contamination. Baran and Gumus Kiral (2021) reported elevated As, Mo, and Sb in Batman soils, with traffic contributing to Sn, Zn, and Pb contamination. Improper storage of mineral oils, batteries, and leaks during industrial production and refining at TPAO, TÜPRAŞ, and BOTAŞ facilities were identified as additional pollution sources. Yildiz and Ozkul (2022) measured As (11.6–90.5 mg kg^−1^), Cr (31.9–1455.2 mg kg^−1^), and Ni (83.1–484.9 mg kg^−1^) in Uşak urban soils, attributing contamination to industrial activities.

Guney et al. (2020) reported severe As (up to 765 mg kg⁻^1^) and Sb (76 mg kg⁻^1^) in Kütahya city due to a geogenic anomaly, posing a serious health risk, while Hg remained low. Comparable As and Sb contamination has been reported in Kraków, Poland (Plak et al., 2024).

Yilmaz et al. (2003) reported elevated Co, Cu, Mn, Pb, and Zn in industrial and urban soils of İzmit, largely due to industrial emissions, waste disposal, and heavy traffic associated with high population density. Odabasi et al. (2010) detected elevated Zn and Pb in Hatay- İskenderun industrial soils relative to rural sites, linked to iron-steel plants, consistent with steel industry markers Zn, Pb, and Cu (Cetin et al., 2007). Simatupang et al. (2024) reported Pb up to 1176 mg kg⁻^1^ in industrial soils of Samut Sakhon, Thailand, highlighting the global relevance of industrial Pb pollution.

Ezer (2009) found Pb and Zn concentrations in Kahramanmaraş roadside soils to be considerably higher in downtown areas, due to traffic density and vehicle repair workshops, with levels positively correlated with traffic volume. Arslan and Gizir (2006) reported elevated Pb, Cd, and Zn in Mersin roadside soils, with the highest Pb concentrations at Çetinkaya Road Station attributed to gasoline vehicle emissions. Cd levels also increased with traffic volume, confirming traffic as the main source, although concentrations generally remained within acceptable limits. Comparable roadside soil contamination has also been reported in Lahore city, Pakistan (Khan & Shah, 2023), Chennai city in India (Gopal et al., 2024), Ibadan city in Nigeria (Akinsete & Olatimehin, 2024), and Bydgoszcz city in Poland (Bartkowiak et al., 2024).

The comparison of PTE concentrations in soils from different Turkish urban locations with national soil quality thresholds is presented in Table 2. These thresholds, defined by the Ministry of Environment and Urbanization of Türkiye (2010), are determined by soil pH, with separate categories for 6 ≤ pH < 7 and 7 ≤ pH < 8. Cd is the most critical: values at the Mersin corridors (~ 125–286 mg kg⁻^1^) are two orders of magnitude above the limits; İzmir, Muğla parks/marketplaces, and Ordu playgrounds also exceed even the higher (1.5 mg kg⁻^1^) threshold, whereas Tuzla (1.16 mg kg⁻^1^) is marginal and Kahramanmaraş (0.003 mg kg⁻^1^) negligible. Pb frequently exceeds 100 mg kg⁻^1^ in Gebze (246 mg kg⁻^1^), Hatay-İskenderun, İzmir, Kahramanmaraş, and Mersin, while Ömerli remains at the lower limit and Tuzla (34 mg kg⁻^1^) remains below. Zn is extreme in industrial and recreational settings, Gebze (632 mg kg⁻^1^), Ordu playgrounds, and Hatay-İskenderun all surpass 200 mg kg⁻^1^; Mersin (~ 156–166 mg kg⁻^1^) typically exceeds the lower but not the upper Zn limit. Cu is generally compliant but breaches the upper guideline in Mersin (≈163–240) and approaches it in Gebze, with several cities near or below the 50 mg kg⁻^1^ threshold. Cr ranges from compliant to extreme: Ömerli exceeds the lower limit; Gebze and parts of İzmir exceed one or both limits; Hatay-İskenderun (798 mg kg⁻^1^) and Denizli (886 mg kg⁻^1^) are far above 100 mg kg⁻^1^. Although no national As standard is found, Kütahya (156 mg kg⁻^1^) indicates strong geogenic enrichment relative to other cities.

**Table 2:** Mean concentrations of PTEs soil pollution (mg kg^−1^) in urban areas for Turkish cities

Cities	PTE (mg kg^−1^)						References
	As	Cd	Cr	Cu	Pb	Zn	
İstanbul. Ömerli	N.A	N.A	83	7.6	71	33	Esen et al. (2024)
İstanbul. Tuzla	N.A	1.16	N.A	50.58	34.29	122.57	Sezgin et al. (2019)
İstanbul. Gebze	9.53	4.41	118	95.88	246	632	Yaylalı-Abanuz (2011)
Batman	22.2	N.A	N.A	39.3	22.9	70.8	Baran and Gumus Kiral (2021)
Bingöl	N.A	0.81	26.88	14.67	30.11	58.42	Vural et al. (2021)
Muğla (main road)	N.A	7.1	N.A	N.A	37.7	N.A	Demirak et al. (2022)
Muğla (park)	N.A	7.5	N.A	N.A	50.2	N.A	
Muğla (marketplaces)	N.A	6	N.A	N.A	91	N.A	
Kütahya	156	N.A	N.A	N.A	N.A	N.A	Guney et al. (2020)
Denizli	N.A	N.A	886	30	17	51	Oudeika et al. (2020)
Ordu (Cumhuriyet playground)	N.A	4.95	49.70	N.A	23.50	566.90	Yesil and Yesil (2019)
Hatay- İskenderun	9	2	798	61	109	531	Odabasi et al. (2010)
İzmir	N.A	26	345	46	133	61	Cetin et al. (2007)
Kahramanmaraş	0.004	0.003	N.A	0.62	242	1.27	Ezer (2009)
Mersin (Train station street)	N.A	125.3	N.A	240.1	174.9	156.6	Arslan and Gizir (2006)
Mersin (Adnan Menderes boulevard)	N.A	286.1	N.A	162.8	330.1	165.7	
Mersin (Ciftlikkoy)	N.A	38.1	N.A	29.3	76.4	79.4	
Ordu (Kemal Şensoy playgroud)	N.A	6.83	54.10	N.A	N.A	330	
İzmir	N.A	< nd	62	35	41	78	Yilmaz et al. (2003)
Eskilehir	11.34	1.37	95.81	33.06	14.34	78.79	Malkoc and Yazici (2017)
Turkish Soil Quality 6 ≥ pH > 7	-	1	60	50	70	150	Ministry of Environment and Urbanization, Türkeye (2010)
Turkish Soil Quality 7 ≥ pH > 8	-	1,5	100	100	100	200	

N.A. no data available.

**Fig. 3:**
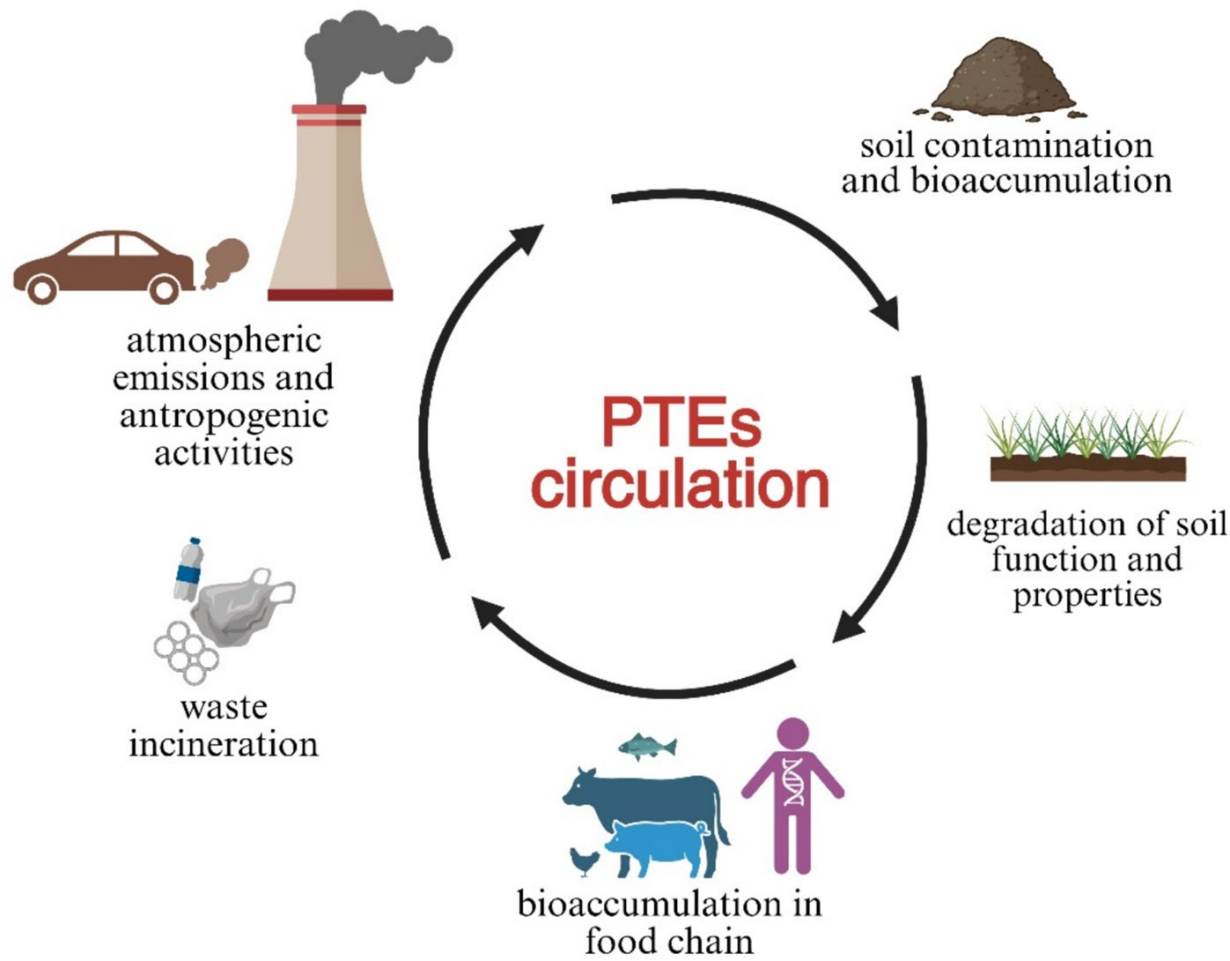
Schematic representation of the PTEs in urban soils, showing their sources, pathways, and bioaccumulation

Taken together, the evidence demonstrates that Cd and Pb represent the most persistent and widespread urban soil contaminants in Türkiye, consistently exceeding national standards and often reaching concentrations comparable to or higher than those in other rapidly industrializing regions. Industrial hubs such as Gebze, Hatay-İskenderun, İzmit, and Denizli have emerge as national hotspots, while elevated Cd and Zn levels in playgrounds (Ordu, Muğla) highlights the public health risks associated with direct exposure in in areas with high child population density. Roadside studies confirm the persistent contribution of traffic emissions to Pb and Cd accumulation, even in cities without major industrial complexes. In contrast, geogenic anomalies, such as the As hotspot in Kütahya, highlight the need for site-specific risk management strategies that distinguish natural enrichment from anthropogenic pollution. Despite the relatively broad geographic coverage of case studies, the literature remains fragmented and inconsistent as most studies measure only total concentrations, with little information on bioavailability, or pollutant interactions (e.g., co-occurrence of PTEs with PAHs and MPs). Moreover, many entries in Table 2 are “N.A.”, reflecting either non-measured pollutants or unreported values, which limits comparability across sites. Finally, mid-sized cities and peri-urban zones remain underrepresented, even though they often experience rapid industrial growth and weak regulatory oversight. Figure 3 presents a schematic illustration of the pathways and cycling of PTEs in urban soils.

### PAHs

**Fig. 4:**
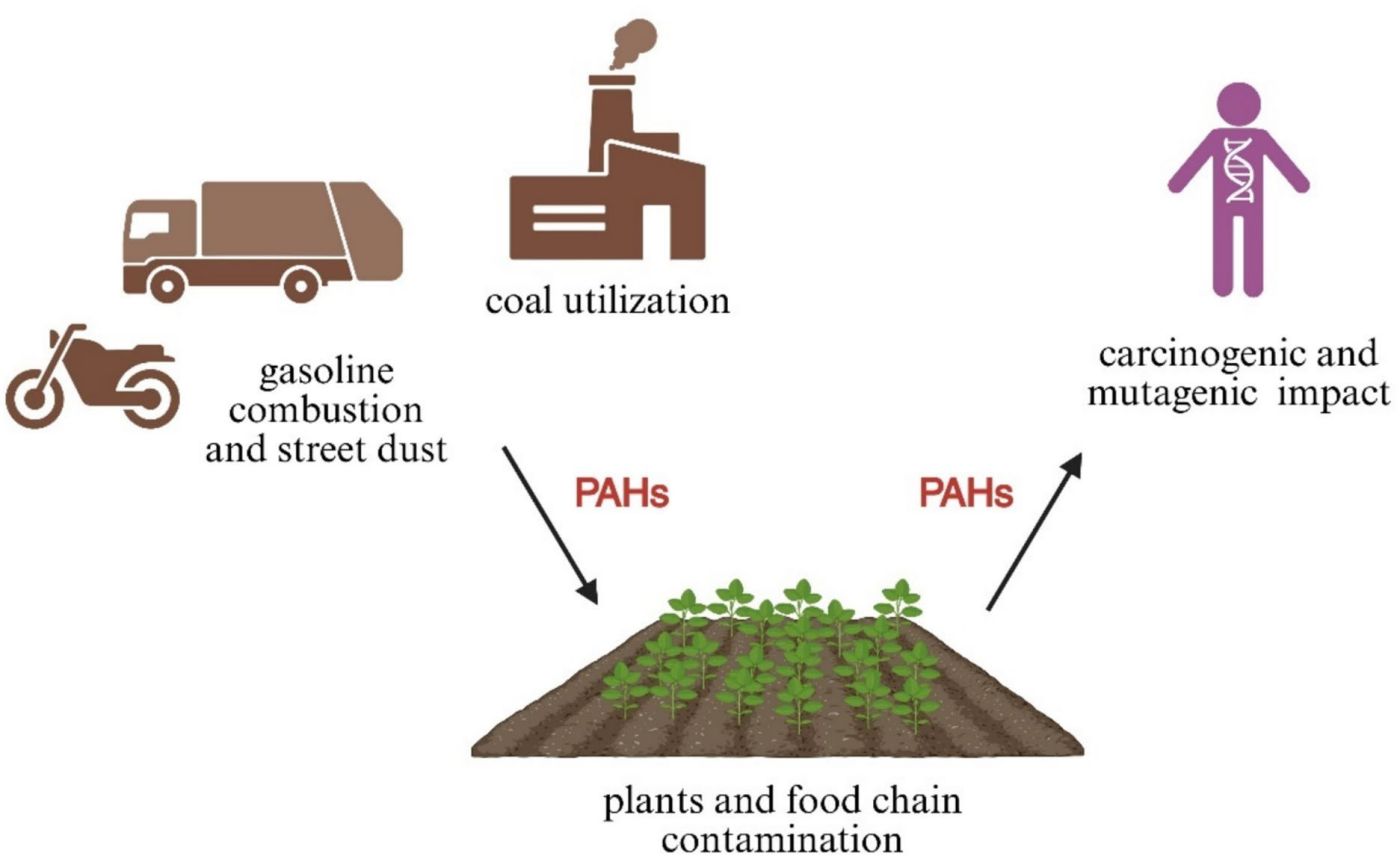
Schematic representation of the sources, pathways, and toxic effects of PAHs in urban soils and food chains

Previous investigations demonstrated the widespread occurrence of PAHs in various environments worldwide, particularly in urban regions adjacent to pollution sources (Akinsete & Olatimehin, 2024; Hümmler et al., 2024; Venkatraman et al., 2024). Among natural reservoirs of PAHs, soils are considered the primary sink, as most PAHs emitted into the environment ultimately accumulate there via atmospheric deposition (Pi et al., 2024; Sung et al., 2024). According to the study by Wild and Jones (1995), around 90% of PAHs are considered to be deposited in soil. However, the concentration and distribution of PAHs vary significantly across regions, influenced by factors such as soil microbial activity, climate conditions and anthropogenic releases (Bandowe et al., 2021). Thus, it is evident that urban soils have 4 to 400 times higher levels of PAHs than soils originating from the natural environment (Sung et al., 2024). Figure 4 schematically illustrates the primary sources, transfer routes, and toxic effects of PAHs identified in urban soils. The pollution of the Turkish urban areas by PAH is presented at Table 3.

**Table 3:** Concentrations of ΣPAHs and their sources in the urban soil in main Turkish cities

Cities	Total PAHs		PAHs Subset	Unit	Source	References
Bursa	13–189		14	μg kg^−1^	vehicle emissions, residential heating emissions	Sanli et al. (2023)
Bursa	8–4970		12	μg kg^−1^	iron–steel and cement factories	Karaca (2016)
İzmir	11–4628		15	μg kg^−1^	steel plants, the petroleum refinery, and the petrochemical plant	Bozlaker et al. (2008)
Dilovası, Kocaeli	49–10,512		15	μg kg^−1^	traffic, iron-steel production and domestic heating and industries	Yurdakul et al. (2019)
İstanbul	113–5537		15	μg kg^−1^	traffic, residential heating, and industrial activities	Cetin et al. (2017)
İstanbul	19–279		16	μg kg^−1^	vehicle emissions, coal combustion	Yukhimets et al. (2020)
İstanbul	Winter	38–5941	8	μg kg^−1^	Traffic, population density, industrial activities	Balcioğlu et al. (2021)
	Spring	15–3389	8			
	Summer	468–927	8			
	Fall	22–427	8			

Maliszewska-Kordybach (1996) classified soil PAH pollution into four categories based on concentration levels: non-contaminated (< 200 μg kg^−1^), weakly contaminated (200–600 μg kg^−1^), contaminated (600–1000 μg kg^−1^), and heavily contaminated (> 1000 μg kg^−1^). Applying this scheme to Turkish urban soils (Table 3), most studies (71%) reported total PAH concentrations exceeding 1000 μg kg^−1^, placing many sites in the heavily polluted category.

Balcioğlu et al. (2021) found that soils from İstanbul’s parks and gardens were heavily contaminated in winter and spring (> 1000 μg kg^−1^), moderately contaminated in summer, and weakly contaminated in autumn (< 600 μg kg^−1^). Diagnostic ratios indicated mainly pyrolytic and mixed pyrolytic-petrogenic sources, influenced by nearby road traffic. Winter maxima were attributed to residential heating (including coal use in some settlements despite widespread natural gas adoption), spring peaks to wet deposition, and summer minima to volatilization and degradation at temperatures approaching 40 °C. These seasonally driven patterns imply time dependent exposure risks in public green spaces. Tanić et al. (2023) reported similar findings in Kruševac, Serbia, where elevated PAH levels occurred in parks and playgrounds near busy streets. Similarly, Akter et al. (2023) reported increased high-molecular-weight (HMW) PAHs in roadside soils in Dhaka, Bangladesh.

In Bursa, Türkiye’s industrial hub, Karaca (2016) observed pronounced spatial contrasts in soil PAHs, with peak concentrations adjacent to busy streets, outdoor grilling areas, and housing. The PAH mixture was dominated by four-ring compounds (fluoranthene, pyrene, benzo[a]anthracene, chrysene), comprising 29–82% of Σ12PAHs, consistent with predominantly pyrogenic sources and a smaller petroleum contribution. Molecular diagnostic ratios revealed that PAH contamination in Bursa soil predominantly originated from pyrolytic sources, with a minor petrogenic contribution. Ambade et al. (2023) likewise reported a dominance of four-ring PAHs in Jamshedpur and Bokaro, East India, attributed to heavy traffic and industrial activities. More recently, Sanli et al. (2023) reported a shift in Bursa toward three-ring PAHs, present in 22.8–57.7% of soil samples; concentrations were lower in summer and higher in winter, and the authors inferred soil to air re-emission during warm periods. A soil–plant transfer assessment also showed elevated PAHs in vegetation, suggesting that plants can act as temporary reservoirs and secondary sources of soils via litterfall or volatilization.

Yurdakul et al. (2019) studied PAH concentrations in soils of the heavily industrialized Dilovası district (Kocaeli), a major industrial center in Türkiye. Concentrations spanned a 214-fold range, indicating strong, ongoing emission sources. PAHs showed clear seasonality, with winter levels approximately twice those in summer. Source apportionment identified traffic, domestic heating, and industrial activities as the main contributors. Similarly, Xie et al. (2024) reported elevated PAHs in urban soils of Handan and Tangshan, China, attributable to traffic and industry. In Istanbul, Yukhimets et al. (2020) observed elevated PAHs around industrial areas on both the European (northwestern) and Asian (southeastern) sides of the city. Acenaphthene and benzo[k]fluoranthene were dominant, with coal combustion and diesel vehicles identified as the primary sources.

Bozlaker et al. (2008) investigated PAH contamination in an industrial area of İzmir. The highest Σ15-PAH concentrations were recorded near Aliağa, where steel plants, a petroleum refinery, and a petrochemical plant are located, indicating these facilities as the primary regional sources. The seasonal variations in soil PAHs were negligible, with the PAH profile being primarily composed of HMW compounds. Chrysene, benzo[g,h,i]perylene, fluoranthene, and pyrene contributed approximately 16%, 13%, 10%, and 9%, respectively, to the Σ15-PAHs. Profiles varied among sites, consistent with differences in source mixtures and distance-dependent atmospheric transport. Odabasi et al. (2010) measured soil PAHs in the Hatay-İskenderun industrial region and found a dominance of medium to HMW compounds. Maximum concentrations were observed north of electric arc furnace and integrated steel plants, consistent with pollutant transport by prevailing southerly winds. Similar industrial signatures have been reported for soils around steel complexes in Canada (Lambert et al., 2011), Poland (Rachwał et al., 2015), and China (Sun et al., 2024).

Cetin et al. (2017) investigated PAH concentrations in İstanbul soils. Concentrations were highest at urban sites adjacent to highways. Across most stations, PAH levels increased as temperature decreased, indicating additional inputs from residential heating. Through factor analysis, sources of contamination were identified as emissions from traffic, combustion of petroleum, coal/biomass, and natural gas, as well as the incineration of medical waste. Fugacity ratio analyses showed temperature and volatility dependent soil-air exchange: soils were prone to re-emit lighter PAHs under warmer conditions while retaining heavier congeners. Toxicological assessment highlighted the severity of PAH pollution in İstanbul soils. Similar source patterns were reported for the Upper Silesian Industrial Region (Poland) by Łyszczarz et al. (2024) and for Chengdu, China by Zhou et al. (2024), where traffic and fossil/biomass combustion elevated urban soil PAHs.

Overall, Turkish urban soils frequently meet the “heavily contaminated” criterion, with industrial zones and traffic corridors as persistent hotspots and winter conditions amplifying burdens. Mixtures are generally pyrogenic and predominantly consist of four-ring compounds, although some cities (e.g., Bursa) exhibit a transition towards three-ring species along with dynamic exchanges between soil and air, as well as soil and plant interfaces.

### Microplastics

While there are still knowledge gaps about the fate and effects of MPs in soils, a growing number of studies are exploring this concern (Akanyange et al., 2021; Akca et al., 2024; Irshad et al., 2024; Jaafarzadeh & Talepour, 2024; Liu et al., 2024). Because soils act as a major reservoir for MPs, their accumulation can alter soil structure, soil biota, and ecosystem functioning (Boots et al., 2019). Reported MP abundances in terrestrial soils are approximately four to twenty-three times higher than those in aquatic systems (Rillig & Lehmann, 2020). MP transport and retention are governed by soil composition and texture, surrounding environmental conditions, and particle size and polymer type (Nematollahi et al., 2020; Wang et al., 2022). For instance, the large pore size and limited water-holding capacity of sandy soil contribute to increased MP retention (Qi et al., 2020). Smaller MPs, measuring less than 1 mm, are more frequently found dispersed within the soil matrix, whereas larger MPs are inclined to stay on the surface of the soil (Alimi et al., 2018; Yu et al., 2022). Upon entering the soil, MPs can engage with other soil components and migrate across the soil profile through different routes such as erosion, leaching, and infiltration (Chukwuemeka et al., 2024; Hartmann et al., 2022). In addition, soil can contribute to the global MPs transport cycle by serving as an atmospheric source of MPs to other reservoirs within the environment via the resuspension of fugitive dust (Zhang et al., 2020). Given the complexity of MP contamination, a comprehensive mitigation strategy is needed to protect human health, ecosystems, and the environment, particularly in urban settings where dense human activity elevates exposure and vulnerability (Wilhelm et al., 2024).

**Fig. 5:**
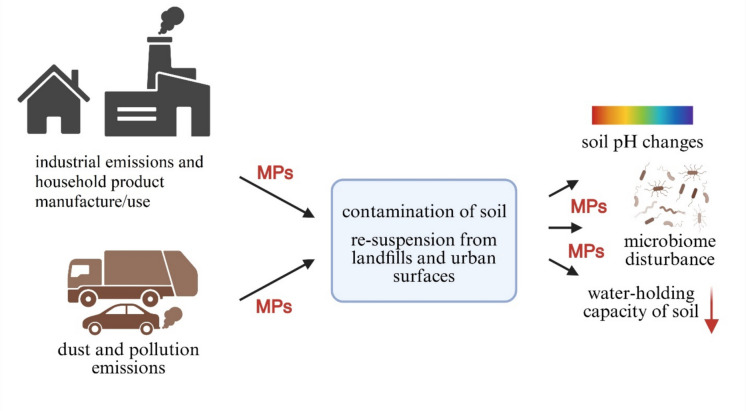
Schematic illustration of MP sources, transport pathways, and impacts on soil properties and microbial communities in urban environments

MPs can enter urban soils through multiple pathways (Fig. 5). These include atmospheric deposition, wind transport, surface runoff, and diverse human activities such as industrial production, urban transportation, synthetic turf/sports-facility flooring, household plastic waste, construction, and agricultural practices (Hu et al., 2024; Luo et al., 2024; Prajapati et al., 2024; Rouhani et al., 2024; Tziourrou & Golia, 2024; Yoon et al., 2024). Over the last decade, research has focused on assessing the prevalence of MPs and their effects on urban soil. However, the investigation related contamination of urban soils by MPs in Türkiye is in the early stages, with only a few papers dedicated to this subject area (Table 4). Türkiye ranks among the top European plastic producers, with an annual production volume of 10.3 million tons (Akca et al., 2024; PAGEV, 2022). The primary sectors of plastic utilization in Türkiye include agriculture, automotive, transport, medical equipment, engineering, and packaging (Akca et al., 2024).

**Table 4:** Pollution by MPs in the urban soil of Türkiye

City	Size	Shape	Polymer type	Color	References
Turkish Mediterranean, Aegean, and Marmara regions	< 1000 μm	Fiber (64.2%)	Polyethylene	Blue	Akca et al. (2024)
İstanbul residential areas	Small-medium	Fragment (%30,56), Pellet (%16,06)	Not reported	Red-Blue	Tunali et al. (2022)
İstanbul recreational areas	Small-medium	Fiber (%36,92), Fragment (30,56)	Polyolefin	Red-Blue	
İstanbul industrial areas	Small-medium	Fragment (%20.44), Fiber (%15,33)	Polyethylene	Yellow-White	
Eskişehir	Not reported	Fiber, Particle	Not reported	Black, Red, Blue, Green, Purple	Buğdaycı and Uygun (2021)

Tunali et al. (2022) investigated MP abundance across industrial, residential, and recreational lands in İstanbul. Residential areas had the lowest MP content (mean = 3,378 items kg⁻^1^), while recreational zones showed the highest abundance (mean = 7,956 items kg⁻^1^), and industrial areas were intermediate, with a mean of 4,488 items kg⁻^1^. Particle-shape distributions differed by land use: microfibers predominated in recreational areas (36.92%), whereas industrial and residential soils were dominated by round/spherical particles (32.85% and 34.26%, respectively). Similarly, Jesus et al. (2024) reported MP contamination in recreational zones of Al Ain (United Arab Emirates), though at lower levels than in İstanbul. Consistent with this pattern, Leitão et al. (2023) reported that natural urban spaces can contain higher MP loads than engineered/impervious spaces. Together, these findings suggest efficient MP transport across urban landscapes and highlight the role of green spaces as effective sinks.

Buğdaycı and Uygun (2021) investigated MP contamination in urban soils in Eskişehir. The analysis revealed that the predominant form was fibers, with black MPs most frequently observed. A significant number of particles occurred along the Kanlıkavak Park pathway in the city center. The authors linked the pollution to high population density and activities that contribute to plastic littering. Consistently, Bhavsar et al. (2024) found that most MPs in Diamond City (India) were fibers (52.90%), and Fernandes et al. (2022) measured high levels of MP fragments and fibers in an urban park in Santo André (Brazil).

Akca et al. (2024) reported MP accumulation in selected agricultural and urban areas across the Mediterranean, Aegean, and Marmara regions of Türkiye. MP concentrations differed significantly by land use, with agricultural areas showing higher levels than urban areas, indicating a strong land-use influence on MP loads. In urban sites, MPs were predominantly blue (54.7%), fibrous (64.2%), < 1000 μm in size (70.6%), and largely polyethylene (78.5%). Because over 90% of the urban sampling locations were parks or roadsides, the authors inferred that urban MP accumulations likely reflect inputs from plastic mulch, plastic infrastructure, litter, and atmospheric deposition. Comparable patterns were reported in Central Arizona and metropolitan Phoenix (USA), where polyethylene accounted for 75% of soil MPs (Chandrakanthan et al., 2024). Similarly, Hu et al. (2024) observed higher MP abundance in agricultural lands than residential areas in Huainan City, China, with polypropylene and polyethylene being dominant.

Across the limited Turkish literature, urban soils consistently contain substantial MP loads, with fibers and polyethylene dominating and green spaces (parks/roadsides) acting as efficient sinks. Land use strongly modulates MP abundance (recreational > industrial > residential in İstanbul), and coarse-textured settings along with wind exposure appear to facilitate MP retention and redistribution. International comparisons (the UAE, the USA, India, Brazil, China) show similar patterns, reinforcing the roles of traffic, recreation, and atmospheric deposition.

## Conclusion and future perspectives

Rapid industrial growth and urbanization have driven strong economic and technological progress in Türkiye, but they have also intensified soil pollution, particularly in major urban areas such as İstanbul. Urban soil quality is fundamental to urban ecosystem functioning and directly affects public health. This review provides a comprehensive assessment of PTEs, PAHs, and MPs in Turkish urban soils over the past two decades, highlighting patterns, sources, and degrees of contamination across 13 cities. The synthesized evidence shows elevated PTE levels, especially for Pb and Cd, which frequently exceeded national/local soil-quality standards, alongside substantial PAH and MP burdens. Despite increasing research, data on PAHs and MPs remain limited. The co-occurrence of these pollutants suggests complex interactions and cumulative effects that may amplify risks, highlighting the need for integrated studies that link urban soil status with environmental-health outcomes.

Although research coverage across Türkiye is relatively broad, existing studies remain fragmented and inconsistent, often limited to total concentrations with limited information on bioavailability or pollutant interactions (e.g., co-occurrence of PTEs with PAHs and MPs). The lack of many unreported or "not given" values limits the potential for comparison of different locations. Additionally, mid-sized cities and peri-urban regions, which often experience quick industrial growth and insufficient regulation, are understudied. Therefore, we recommend the following priorities: (i) a harmonized national monitoring program with common analyte lists, standardized units, and consistent land-use metadata; (ii) the adoption of risk-relevant metrics; (iii) seasonal sampling and source apportionment to distinguish among traffic, industry, and heating contributions; (iv) targeted management in public green spaces frequently used by children and industrial/roadside corridors; and (v) open data infrastructure to enable cross-city comparisons and trend analysis. Addressing these priorities will strengthen regulation and remediation, protect public health, and support sustainable urban development in Türkiye. 

## Data Availability

No datasets were generated or analysed during the current study.
